# Associated SNPs, Heritabilities, Trait Correlations, and Genomic Breeding Values for Resistance in Snap Beans (*Phaseolus vulgaris* L.) to Root Rot Caused by *Fusarium solani* (Mart.) f. sp. *phaseoli* (Burkholder)

**DOI:** 10.3389/fpls.2021.697615

**Published:** 2021-09-28

**Authors:** Abigail R. Huster, Lyle T. Wallace, James R. Myers

**Affiliations:** ^1^Department of Horticulture, Oregon State University, Corvallis, OR, United States; ^2^USDA-ARS, Plant Germplasm Introduction and Testing Research Unit, Washington State University, Pullman, WA, United States

**Keywords:** common bean, disease resistance, genome wide association studies, genomic prediction, best linear unbiased prediction, root morphology, genomic selection

## Abstract

Root rot is a major constraint to snap bean (*Phaseolus vulgaris*) production in the United States and around the world. Genetic resistance is needed to effectively control root rot disease because cultural control methods are ineffective, and the pathogen will be present at the end of one season of production on previously clean land. A diversity panel of 149 snap bean pure lines was evaluated for resistance to *Fusarium* root rot in Oregon. Morphological traits potentially associated with root rot resistance, such as aboveground biomass, adventitious roots, taproot diameter, basal root diameter, deepest root angle, shallowest root angle, root angle average, root angle difference, and root angle geometric mean were evaluated and correlated to disease severity. A genome wide association study (GWAS) using the Fixed and random model Circulating Probability Unification (FarmCPU) statistical method, identified five associated single nucleotide polymorphisms (SNPs) for disease severity and two SNPs for biomass. The SNPs were found on Pv03, Pv07, Pv08, Pv10, and Pv11. One candidate gene for disease reaction near a SNP on Pv03 codes for a peroxidase, and two candidates associated with biomass SNPs were a 2-alkenal reductase gene cluster on Pv10 and a Pentatricopeptide repeat domain on Pv11. Bean lines utilized in the study were ranked by genomic estimated breeding values (GEBV) for disease severity, biomass, and the root architecture traits, and the observed and predicted values had high to moderate correlations. Cross validation of genomic predictions showed slightly lower correlational accuracy. Bean lines with the highest GEBV were among the most resistant, but did not necessarily rank at the very top numerically. This study provides information on the relationship of root architecture traits to root rot disease reaction. Snap bean lines with genetic merit for genomic selection were identified and may be utilized in future breeding efforts.

## Introduction

Root rot is a serious disease that affects common beans (*Phaseolus vulgaris*) wherever they are grown. It has been and continues to be a primary yield limitation in both snap and dry bean production. Root rot is a broad term that can refer to infection by a variety of pathogens or complexes thereof (Abawi et al., 1985). The most serious and widespread causal pathogen, *Fusarium solani* f. sp. *phaseoli*, has been reported to cause yield losses of up to 84% in the United States (Schneider et al., 2001). This organism is the primary, although not necessarily exclusive, root rot pathogen in Oregon snap bean fields. There is currently no satisfactory management technique to control root rot in snap beans with cultural and chemical methods having met with limited success (Burke and Miller, 1983). The best cultural option available to control root rot is crop rotation but the four-to-five-year interval that is required is impractical for most farmers. With so few options, genetic resistance is of paramount importance. The benefits of resistance extend beyond mitigating disease. Without functional root systems, it is impossible to select for other traits, such as abiotic stress and nutrient use efficiency that are needed to combat climate change and adapt to agricultural intensification.

Most prior genetic analyses of *F. solani* root rot resistance have been conducted with biparental dry bean populations (Supplementary Table 1). Many were conducted with RAPD marker systems that are difficult to rectify with contemporary SNP-based maps (Chowdhury et al., 2002; Román-Avilés and Kelly, 2005; Navarro et al., 2008; Schneider et al., 2001). SNPs have become the preferred marker for linkage and association mapping because of their abundance, repeatability and reference to physical location within the genome (Blair et al., 2013; Cortés et al., 2011). Unlike others who focused exclusively on dry beans, Navarro et al. (2008) and Hagerty et al. (2015) used snap x dry bean populations to map QTL for root rot resistance. In all cases, resistance was inherited quantitatively with one to 15 QTL explaining from five to 53% of total phenotypic variance. Where reported, heritabilities have ranged from 10 to 99%, with the majority being in the low to moderate range. One genome wide association study (GWAS) has been conducted in dry bean for resistance to *F. solani* root rot. This study identified SNP associations in Andean and Middle American diversity panels (Zitnick-Anderson et al., 2020). They found sixteen unique SNP associations in an Andean diversity panel on Pv01, Pv02, Pv03, Pv04, Pv07, Pv08, Pv09, and Pv11, and seven unique SNP associations in a Middle American panel on Pv01, Pv03, Pv04, Pv07, and Pv08 (Zitnick-Anderson et al., 2020). Further GWAS studies have been conducted on root rot caused by *Pythium* spp., *Pythium ultimum*, *Fusarium oxysporum* and *Rhizoctonia solani* in dry bean (Oladzad et al., 2019; Dramadri et al., 2020; Diaz L. M. et al., 2021; Paulino et al., 2021). With *F. solani*, the studies listed in Supplementary Table 1 have not found major QTL associated with resistance and the general consensus is that resistance is conditioned by several to many genes with small individual effect.

There is evidence that the genetic background of snap beans has unique characteristics which warrants examination on its own (Wallace et al., 2018). In particular, the genetic background of snap beans is highly mixed between the Andean and Middle American gene pools with unknown effects on the interactions of genes. Moreover, snap beans have been selected for succulent, low fiber pods mostly in isolation from dry beans since their assimilation by Europeans starting in the 1500’s and this time frame may have been sufficient for unique resistance traits to evolve within snap beans.

The traditional GWAS model is a mixed linear model with a correction for kinship and population structure that adequately controls type I statistical errors. Last-generation GWAS models, such as FarmCPU, have improved sensitivity and statistical power with similar control of type I statistical errors and much improved control of type II statistical errors (Liu et al., 2016; López-Hernández and Cortés, 2019). Work on last-generation GWAS models (FarmCPU, BLINK, and SUPER) indicates that they are comparable and complement with each other when used in parallel, although subtle differences have been found, such as non-redundant results (FarmCPU) or a greater number of associated SNPs (BLINK) in a study of heat stress in common bean (López-Hernández and Cortés, 2019). Both BLINK and FarmCPU iteratively utilize a random and fixed model and may have an advantage over SUPER in having a lower type II statistical error rate (López-Hernández and Cortés, 2019).

Marker assisted selection (MAS) have been most successfully applied to traits conditioned by major genes, or in some cases, major QTL (Assefa et al., 2019) and specifically in breeding programs to introgress disease resistance. Over 40 SCAR or SRAP markers linked to resistance to 11 pathogens are available in common bean (BIC, 2021b). Only two of these are for root pathogens (*Fusarium oxysporum* and *Pythium ultimatum*), where resistance is conditioned by major genes. Some studies on *F. solani* resistance indicate that the markers that were discovered may be useful in breeding for resistance. However, there is little evidence of their application in breeding programs. The underlying reason for this is probably the polygenic nature of *F. solani* resistance. MAS has not proven to be very effective for such traits. Genomic selection (GS) is emerging in common bean as a technique that allows selection of quantitative traits without the labor-intensive approach that traditional MAS would require (Assefa et al., 2019). GS models generally use many markers distributed across the genome, and as a result, are more effective than traditional MAS in selection for traits with many genes with small effect. GS has been applied to common bean for root rot (Diaz L. M. et al., 2021) as well as to agronomic traits (Keller et al., 2020), cooking time (Diaz S. et al., 2021), and nematode resistance (Wen et al., 2019; Shi et al., 2021) to discover genotypes with the best breeding values for recombination schemes, but deployment in breeding programs is only beginning.

Differing models for genomic selection are similar in their predictive accuracy. One study of maize traits found that rrBLUP had a slightly higher predictive accuracy in comparison to four other genomic prediction models (Riedelsheimer et al., 2012). Other research into genomic selection models in barley and wheat found no differences, but a study of loblolly pine found rrBLUP lacking when applied to oligogenic traits with a few major genes (Heslot et al., 2012; Resende et al., 2012).

The purpose of this research was to improve the understanding of the genetics underlying resistance to *F. solani* sp. *phaseoli* in snap beans under field conditions typically found in a major snap bean growing region of the United States. As the genetic background of snap beans is unique, this is an important gap that needs investigation separate from previous dry bean studies of *Fusarium* root rot genetic architecture. To achieve this goal, three research focus areas were identified: (1) Analysis of root and plant morphological traits in a diversity panel of snap beans as related to root rot resistance or susceptibility, (2) GWAS on root rot resistance in a diversity panel of snap beans, and (3) Genomic prediction of cultivars to identify lines with superior breeding potential based on the totality of all marker effects in order to better capture minor allelic effects that may be missed by GWAS.

## Materials and Methods

### Study Site and Experimental Design

In this study, 149 pure lines of the Common Bean Coordinated Agricultural Project (BeanCAP) Snap Bean Diversity Panel (SBDP; see data availability statement for details on this panel) were evaluated for resistance to root rot, which primarily consists of *F. solani* in Oregon. This diversity panel contains pure line examples of both centers of domestication with a representative cross section of historical and contemporary snap beans, but no wild materials. About 83% of the lines in the SBDP are of Andean center of domestication with the remainder being of Mesoamerican derivation (Wallace et al., 2018). They can be further classified into eight groups based on Structure analysis, with some lines having genetic contributions from as many as seven groups. Since snap beans have undergone a high level of intermixing relative to dry beans between the centers of domestication (Wallace et al., 2018), more than 50% of the snap beans in the panel contain some genetic background from both centers of domestication.

Strongly root rot susceptible (‘Seabiscuit’, ‘Shade’, and ‘Zodiac’) and strongly resistant (‘Black Valentine’, ‘Impact’, and ‘Widusa’) cultivars were included. The OSU cultivars included in the panel were bred and selected on the research farm under constant root rot pressure, and as a result, have high levels of resistance, and consistently grouped with the most resistant lines in the diversity panel. Additionally, the panel included ‘FR-266’, an experimental snap bean line bred in the Pacific Northwest for *F. solani* root rot resistance (Silbernagel, 1987). This line has been used in biparental mapping population studies of root rot resistance (Schneider et al., 2001). It has been a check in our root rot breeding nursery trials, where it shows moderate levels of resistance. The complete panel was used, except for ‘BBL 274’, which was unavailable for planting. In late spring of 2014 and 2015, four replicates of the SBDP were planted at the Oregon State University Vegetable Research Farm. The Vegetable Research Farm is located in Corvallis, Oregon on Chehalis silty clay loam soil at latitude N44.571209, longitude W123.243261 at 77 masl. The studies took place in our root rot “purgatory plot” that had been planted continually with snap beans for over 25 years in an effort to build a heavy pathogen population and increase disease pressure for more effective screening. In monitoring of bean root pathogens present at the Vegetable Research Farm, we have always found *F. solani* to be the primary pathogen (see Cirak and Myers, 2021 for latest assay). To further encourage heavy and uniform disease pressure, the trials were well irrigated (2.5 cm of water weekly by solid set sprinklers) in the beginning of each season, as high soil moisture levels aid in infection. After pod set, irrigation was reduced to increase abiotic stress levels. The late season irrigation schedules were determined based on weather conditions.

The trials were planted with a modified randomized complete block design with the field divided into four replicated blocks on a north-south axis. This method of blocking was chosen as the size of this experiment exceeded previous years’ plantings and extended into soil that may have had a lower level of disease pressure. Due to their unique characteristics and need for a trellis system, the pole beans were planted in a separate four block randomization at the west end of the field. The plots were 3.0 m long, planted in a single row at a density of 50 seeds per plot. Rows were spaced 75 cm apart. A border row of OSU5446, a root rot susceptible experimental line, was planted on the north and south edges of the field, as well as 1.5 m end plots on the east and west ends of each row to minimize edge effects. Planting dates were 10 June in 2014 and 21 May in 2015. The seed was treated with captan pre-emergent fungicide (Bonide Products Inc.) prior to planting to improve germination and emergence uniformity and reduce differences in stand among lines.

### Field Evaluation

Data collection began when the earliest lines were at 50% buckskin pod stage (when half the pods per bush have lost their chlorophyll and have taken on a flexible, leathery texture). Each plot was evaluated at this uniform phenological stage. A Shovelomics protocol (Lynch and Brown, 2001, 2013) was used to perform evaluations. The SBDP was evaluated for several morphological traits including taproot diameter, largest basal root diameter, deepest and shallowest basal root angles, and aboveground biomass to investigate correlations between plant structure and disease resistance. Five consecutive plants from the center of the plot were dug with a 30 cm radius of soil around the roots, and carefully shaken and washed to remove the soil without damaging the roots. The five plants were evaluated on a 1–5 (1 = least and 5 = most biomass) scale as a single unit for aboveground biomass (Supplementary Figure 1). A subsample of two randomly selected plants from the original five were evaluated independently for taproot diameter, largest basal root diameter, deepest basal root angle, shallowest basal root angle, adventitious roots (1–3 scale; 1 = few, 3 = many roots), and disease severity (1–5 scale; Table 1). In evaluating disease severity of *F. solani*, nearly all researchers have used 1–5 or 1–9 visual rating scales (Azzam, 1956; Baggett et al., 1965; Abawi, 1990; Hagerty et al., 2015; BIC, 2021a). Taproot and largest basal root diameter were recorded with digital calipers. The measurements were taken 1 cm. below where the root emerged from the hypocotyl. The deepest (closest to the taproot) and shallowest (closest to the soil line) basal root angles were measured by laying the specimen on a cutting board marked with protractor angle increments (Supplementary Figure 2).

**TABLE 1:** Scale for rating *Fusarium solani* root rot symptoms in the BeanCAP Snap Bean Diversity Panel grown at the Oregon State University Vegetable Research Farm for a genome wide association study.

Score	Root rot rating scale description
1.0	Clean white root
1.5	Few external red or brown lesions
2.0	Some external lesions, but root still firm and white inside
2.5	Some external lesions, red discoloration of pith, but root is firm
3.0	Significant external infection, red to brown pith
3.5	Spongy brown lesions are present
4.0	Root is soft and rotten
4.5	Root is very rotten, falling off
5.0	Root is absent, plant ends in rotten stump

Root angle difference, root angle average, and root angle geometric mean were calculated from deepest and shallowest root measurements. Root angle difference was the shallowest root angle subtracted from the deepest root angle. This conveys the span of the soil profile accessed by the plant. Root angle average is the mean of the deepest and shallowest root angles and expresses the general orientation of the roots, from zero to 90°. Root angle geometric mean is the geometric mean of the root angle average and the root angle difference and was formulated to provide a single value that integrated soil profile span and root orientation.

### Statistical Analysis of Field Trials

To characterize the variation observed in the 2014 and 2015 trials, the following statistical approach was used. First, homogeneity of variances across years was examined using PROC GLIMMIX (SAS version 9.3: SAS institute, Cary, NC) using the model [Trait] = Variety Rep(Year) Year Variety^∗^Year with Year treated as a random effect and the Covtest option to test for homogeneity of variances. Variances from 2014 and 2015 demonstrated homogeneity, and both years of data were combined into a single analysis. Second, normality by year was examined using PROC GLM with the model [Trait] = Rep Variety. Third, a mixed model analysis of variables with years combined was performed using PROC GLM with the model [Trait] = Variety Year Rep(Year) Year^∗^Variety with Year, Rep(Year) and Year^∗^Variety treated as random effects. As the two individual plants measured from each plot were intended to capture information on a plot-mean basis rather than an individual plant basis, mean scores for each plot were used.

### Multiple Correlation Analysis Among Traits

To evaluate whether root morphological traits and disease severity were positively or negatively associated, a Pearson’s correlation coefficient analysis was performed in SAS 9.3 on the least square means of the phenotypic data for disease severity, aboveground biomass, adventitious roots, basal root diameter, taproot diameter, shallowest root angle, deepest root angle, root angle difference, root angle average, and root angle geometric mean. Least square means were generated from combined data from the 2014 and 2015 trials when ANOVAs were conducted as described above. Correlations were generated for all pairwise combinations of traits.

### Genotyping

The genotypic dataset consisted of 10,607 SNPs generated by using two Illumina iSelect 6K Gene Chip sets (BARCBEAN6K_1 and BARCBEAN6K_2) (Song et al., 2015). These BeadChips were designed following sequencing a diverse set of 17 dry bean cultivars with 10 from the Mesoamerican and seven from the Andean centers of domestication. SNPs with 50% or greater missing data were discarded (Song et al., 2015). Remaining missing genotypes were imputed using fastPHASE, which uses the Hidden Markov Model to indicate the cluster membership of haplotypes (Scheet and Stephens, 2006). Genotypic data for the ‘Panama’ genotype was unavailable and was excluded from the GWAS and BLUP analysis.

### Heritability

Narrow sense and broad sense heritability are essentially equivalent in a highly inbred crop such as common bean. With complete homozygosity, it can be assumed that there are no dominance effects present. In the absence of dominance effects, variance among inbred lines, or Var(G), provides an estimate of additive genetic variance or Var(A), rendering the two equations equivalent (Hallauer et al., 2010). Additive x additive epistasis may inflate estimates of narrow sense heritability, but is typically minimal in a diploid crop such as common bean. The formula:

$${\hat{h}}^{2}=\frac{{\hat{\sigma }}_{g}^{2}}{\frac{{\hat{\sigma }}^{2}}{re}+\frac{{\hat{\sigma }}_{ge}^{2}}{e}+{\hat{\sigma }}_{g}^{2}}$$

was used to determine heritability, where ${\hat{\sigma }}_{g}^{2}$ is the estimated genotypic variance component, ${\hat{\sigma }}_{ge}^{2}$ is the estimated genotype by environment interaction variance component, ${\hat{\sigma }}^{2}$ is the estimated experimental error variance, *e* is the number of environments, and *r* is the number of replications per environment. Heritability for each trait was calculated using SAS code developed by Holland et al. (2003). Mixed model analysis (PROC MIXED, SAS 9.3) was used to obtain variance components. Variance components were estimated using the restricted maximum likelihood (REML) method. All model components were treated as random effects. Heritability was calculated on a line mean basis.

### Genome-Wide Association Study

The entire SNP dataset was utilized for GWAS analysis. The phenotypic data used for GWAS was a single value for each trait, averaged across four reps and two years. Due to the incongruity of a pole bean plant architecture for biomass measurements, pole type beans were removed from the biomass analysis leaving 139 genotypes (lines) for this analysis. All other traits were measured with the full set of genotypes.

The FarmCPU statistical method was performed in version 4.0.2 of the R software environment (Liu et al., 2016). To derive SNP *R*^2^ values, FarmCPU was run within GAPIT (version 3) with the added code, Random.model = TRUE. The SNP data was formatted in Microsoft Excel and was filtered for a minor allele frequency (MAF) of 0.05 within *R*.

The principal component analysis (PCA) was conducted in TASSEL, version 5.2.73.^1^ Principal components one to five accounted for 22, 33, 41, 48, and 52% of the variation, respectively. Based on the widely accepted criterion of principal components accounting for between 25 and 50% of the variation (Oladzad et al., 2019; Zitnick-Anderson et al., 2020), the choice of principal components was narrowed to between two and four. To further narrow the choice of principal components, QQ plots were examined for fit around the null distribution to make the final selection of two principal components (Supplementary Figure 3). Linkage Disequilibrium (LD) heat maps for individual chromosomes were also generated in TASSEL using the full matrix in lieu of the sliding window.

Two different thresholds were examined for a cutoff of significance in the Manhattan plots. The more conservative threshold was a Bonferroni cutoff that utilized the effective marker number of 2,411 as determined by the SimpleM method (Gao et al., 2010). This generated an alpha 0.05 threshold of 4.68 as expressed as a negative log value. In addition, a 10,000 bootstrap threshold was generated for an alpha of 0.05 (Mamidi et al., 2014). This bootstrap identified a threshold of 4.51 negative log.

### Candidate Gene Search

Associated SNP positions were located in the common bean genome as shown in the Phytozome JBrowse genome browser (Phytozome, version 12.1; *P. vulgaris* genome, version 2.1). Using conservative estimates of linkage disequilibrium in common bean (Soltani et al., 2018; Oladzad et al., 2019) and in consideration of the fact that no wild materials are included in our panel, we chose to bracket a region of ±100 kb in our search for candidate genes. Each gene model within the bracketed region was researched for its potential role in disease resistance or biomass.

### Genomic Prediction

Genomic estimated breeding values were calculated by adding the fixed effect BLUE value for a given trait to the random effect BLUP value for a given bean line and trait as determined by the rrBLUP R package (Endelman, 2011). rrBLUP is equivalent to gBLUP when QTLs are many, there are no major QTLs and QTLs are evenly distributed across the genome (Bernardo, 2020). They differ in that rrBLUP calculates SNP effects from a set of related individuals whereas gBLUP uses markers to estimate relatedness among individuals. Genomic prediction utilized the entire SNP dataset.

To evaluate the predictive power of the rrBLUP calculations, cross validation was performed by randomly splitting all the genotypes within this study into a training set and validation set. The models evaluated used ratios of training set to validation set of 60:40, 70:30, 80:20, and 90:10%. Random partitioning into training and validation sets with the training set used in rrBLUP to predict the phenotype of the validation set was iterated 10,000 times (utilizing all SNPs) with 10 repetitions at each level for each trait from which the mean predictive accuracy (*r*) was determined. Correlations between observed and predicted values using the entire population (100%) in both the rrBLUP calculations and the cross validated rrBLUP calculations were determined in R using a Pearson correlation coefficient.

Associated SNPs from the GWAS analysis were not added to the rrBLUP model as fixed effects (Spindel et al., 2016) because of the relatively low *R*^2^ values of variance explained by associated SNPs but we did investigate the effect of number of SNPs retained in the model on prediction accuracy. SNPs were sorted from lowest to highest *P* value. From these, nine subsets (in addition to the full set) were created. The full SNP sets had 7,082 for biomass and 8,032 for all other traits (number of SNPs retained after filtering for MAF < 0.05). These were reduced in an exponential manner (3,541, 1,770, 885, 442, 221, 120, 55, 28, and 14 SNPs for biomass and 4,018, 2,009, 1004, 502, 251, 126, 63, 32, and 16 for all other traits) to create the subsets. Each subset contained the most highly significant SNPs identified by GWAS. For each subset, the correlation of observed with predicted values was computed in rrBLUP.

## Results

### ANOVA

Means and standard errors for the traits measured in the BeanCAP SBDP are shown in Table 2. Histograms (Supplementary Figure 4) based on LSMeans showed traits to be approximately normal in distribution except for biomass. Biomass was unimodal but right skewed for LSMeans. The lines making up the BeanCAP SBDP exhibited large differences for all of the traits evaluated. Mean squares for the ANOVA model were highly significant for all traits evaluated (Table 3). Mean squares for lines were either significant or highly significant for all traits evaluated, with lower significance levels corresponding to the root angle measurements and the traits derived thereof. Mean squares for replicate were either significant or highly significant, except for the derived trait root angle difference. The mean square for year was significant for taproot diameter, basal root diameter, shallowest root angle, deepest root angle, root angle average, and root angle geometric mean. It was not significant for any other traits. In no cases were years highly significant. Year by line interaction was significant for disease, basal root diameter, deepest root angle, and root angle geometric mean. It was highly significant for aboveground biomass and adventitious roots (Table 3).

**TABLE 2:** Means and standard error (SE) (*N* = 16), and narrow sense heritability (*h*^2^) and 95% confidence intervals for heritability for *Fusarium solani* root rot symptoms (disease severity), plant biomass and root parameters of lines grown in the BeanCAP Snap Bean Diversity Panel at the Oregon State University Vegetable Research Farm in 2014 and 2015.

Trait	Mean^1^	SE (mean)	*h* ^2^	95% Confidence interval (*h*^2^)
Disease severity	3.10	0.01	0.74	(0.66–0.82)
Aboveground Biomass	3.28	0.02	0.75	(0.67–0.83)
Adventitious Roots	1.98	0.02	0.64	(0.52–0.76)
Taproot Diameter (cm)	2.27	0.02	0.51	(0.35–0.67)
Basal Root Diameter (cm)	2.08	0.02	0.47	(0.29–0.64)
Shallowest Root Angle	16.14	0.30	0.38	(0.18–0.58)
Deepest Root Angle	55.68	0.31	0.38	(0.18–0.58)
Root Angle Average	39.55	0.36	0.41	(0.22–0.60)
Root Angle Difference	35.91	0.25	0.32	(0.10–0.54)
Root Angle Geometric Mean	36.10	0.23	0.33	(0.12–0.55)

*^1^Disease severity rated on a 1–5 scale where 1 is resistant and 5 is susceptible; Biomass rated on a 1–5 scale where 1 is the least and 5 the most biomass accumulation; Adventitious roots rated on a 1 – 3 scale where 3 = most adventitious roots; and root angle measurements are in degrees from 0° to 90° where 0° represents a horizontal position.*

**TABLE 3:** Degrees of freedom, mean squares, and significance level for model, year, bean line, replicate within year, and year by line interaction from an analysis of variance for traits associated with *Fusarium solani* disease reaction and plant and root parameters evaluated in trials at the Oregon State University Vegetable Research farm near Corvallis, of the BeanCAP Snap Bean Diversity Panel in 2014 and 2015.

Source of variation	d.f.	Disease severity	Aboveground biomass	Adventitious roots	Taproot diameter	Basal root diameter
**Disease, plant, and primary root traits**						
Model	301	0.80***	2.40***	1.00***	0.77***	0.58***
Year	1	2.03^*ns*^	10.80^*ns*^	13.04^*ns*^	21.51*	22.46*
Line	147	1.25***	3.71***	1.33***	0.89***	0.62***
Rep(Year)	6	1.47***	4.97***	3.66***	2.58***	1.99***
Year*Line	147	0.32*	0.92***	0.48***	0.43^*ns*^	0.33*
*R* ^2^		0.55	0.58	0.54	0.42	0.44
CV		15. 4	23.4	27.1	26.6	24.2

**Source of variation**	**d.f.**	**Shallowest root angle**	**Deepest root angle**	**Root angle difference**	**Root angle average**	**Root angle geometric mean**

**Derived root traits**						

Model	301	186.4***	210.0***	240.0***	138.0***	103.8***
Year	1	5455.4*	5101.0*	6.2^*ns*^	5276.6*	1242.2*
Line	147	200.6*	233.2*	285.2*	145.4***	118.6*
Rep(Year)	6	494.0***	418.5*	241.4^*ns*^	395.8***	155.7*
Year*Line	147	123.8^*ns*^	145.0*	196.2^*ns*^	85.2^*ns*^	79.2*
*R* ^2^		0.35	0.38	0.32	0.38	0.36
CV		68.1	19.4	33.0	24.3	22.0

*Shown at the bottom of the table are *R*^2^ values and coefficient of variation values. *R*^2^ is the regression coefficient for fit to the general linear model. ns = not significant; ^∗^ = significant at *P* < 0.05; ^∗∗∗^ = significant at *P* < 0.001.*

### Multiple Correlation Analysis Among Traits

Disease severity was negatively correlated with aboveground biomass, basal root diameter, and taproot diameter (Table 4), and positively correlated with adventitious roots, shallowest root angle, and deepest root angle. Aboveground biomass, basal root diameter and taproot diameter were highly positively correlated (Table 4). Aboveground biomass and taproot diameter were negatively correlated with shallowest and deepest root angle. Basal root diameter showed the same negative relationship with shallowest root angle but did not have a significant correlation with deepest root angle. Shallowest and deepest root angles were positively correlated with each other.

**TABLE 4:** Pearson multiple correlation coefficients^1^ for Least Square Means of the BeanCAP Snap Bean Diversity Panel evaluated for *Fusarium solani* disease and plant and root traits at the Oregon State University Vegetable Research Farm near Corvallis in 2014 and 2015.

	Biomass	Adventitious roots	Basal root diameter	Taproot diameter	Shallowest root angle	Deepest root angle	Root angle difference	Root angle average	Root angle geometric mean
Disease severity	−0.35***	0.26**	−0.27**	−0.40***	0.37***	0.32***	−0.02^*ns*^	0.42***	0.19*
Biomass		0.13^*ns*^	0.21*	0.19*	−0.37***	−0.18*	0.14^*ns*^	−0.33***	−0.04^*ns*^
Adventitious roots			−0.09^*ns*^	−0.24**	0.03^*ns*^	0.13^*ns*^	0.09^*ns*^	0.10^*ns*^	0.13^*ns*^
Basal root diameter				0.37***	−0.17*	−0.05^*ns*^	0.10^*ns*^	−0.13^*ns*^	0.03^*ns*^
Taproot diameter					−0.23**	−0.46***	−0.23**	−0.43***	−0.39***
Shallowest root angle						0.34***	−0.53***	0.80***	0.00^*ns*^
Deepest root angle							0.62***	0.83***	0.93***
Root angle difference								0.08^*ns*^	0.84***
Root angle average									0.59***

^1^Probability > | r| under H_*o*_: *Rho* = 0. * = significant at *P* < 0.05; ** = significant at *P* < 0.01; and *** = significant at *P* < 0.0001. ^*ns*^ = not significant.

### Heritability

A range of heritabilities was observed for the different traits measured (Table 2). Aboveground biomass and disease severity had the highest heritability with *h*^2^ = 0.75 and 0.74, respectively. The root angle traits had the lowest heritability, ranging from *h*^2^ = 0.32 for root angle difference to *h*^2^ = 0.41 for root angle average.

### Genome-Wide Association Study

GWAS assumes normality (Goh and Yap, 2009). The disease severity and biomass datasets were normally distributed based on QQ plots of residuals generated from an ANOVA analysis of years, reps, and genotypes. The tap root diameter and basal root diameter datasets were also normally distributed for residuals after a square root transformation. Adventitious roots, short root angle, and deep root angle could not be made to conform to normality for their residuals. A GWAS analysis was conducted on all datasets, including square root transformed tap root diameter and basal root diameter. GWAS analysis of tap root diameter, basal root diameter, adventitious roots, short root angle, and deep root angle did not generate any significant SNP associations with a two PCA FarmCPU model.

A biplot of the first two PC axes (Supplementary Figure 3) revealed a clinal gradient along PCA 1 for center of domestication, with those lines clearly from the Mesoamerican center of domestication having strong positive scores and those with Andean background ranging from positive to negative scores. PCA 2 primarily separated European-bred small sieve cultivars from blue lake and pole bean types, but without discernable differentiation for Andean background cultivars. These results generally match our findings with Structure analysis (Wallace et al., 2018).

**FIGURE 1:**
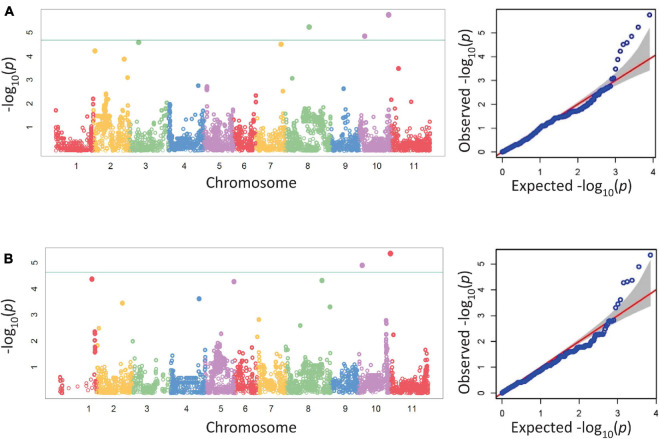
Manhattan and corresponding Q-Q plots from a GWAS analysis of disease severity **(A)** and biomass **(B)** in the BeanCAP Snap Bean Diversity Panel evaluated in 2 years for *Fusarium solani* reaction at the Oregon State University Vegetable Research Farm. The Bonferroni cutoff based on effective marker number (-log_10_ 4.68, α = 0.05) is shown as a solid line. For the Q-Q plots, the null distribution is shown as a red line.

Five SNPs were associated with disease severity on chromosomes Pv03, Pv07, Pv08, and Pv10 with two SNPs on Pv10 (Table 5 and Figure 1). SNPs ss715639797, ss715649485, and ss715646318 on Pv08 and Pv10 were identified through a Bonferroni threshold. A further two SNPs, ss715647578 and ss715646526, were identified on Pv03 and Pv07 through a bootstrap analysis. The phenotypic variation (*R*^2^) explained by SNPs indicated a low contribution to disease resistance by each SNP ranging in value from 0.9 to 10.8% with the highest value for ss715647578 on Pv03 and the lowest value for ss715646526 on Pv07. The effect of allelic substitution was negative for three SNPs and positive for two (Table 5). Effect was relatively small with a cumulative effect of altering disease severity score by 0.5. Two SNPs were associated with biomass on chromosomes Pv10 and Pv11 (Table 5 and Figure 1). SNPs ss715649390 and ss715645486 on Pv10 and Pv11, respectively, were identified through a Bonferroni threshold. No further SNPs were identified through a bootstrap analysis. The *R*^2^ values were 11.3% for ss715645486 and 14.8% for ss715649390, and the former had an allelic substitution effect of -0.12 while the latter had a relatively larger effect of -0.18 (Table 5). The cumulative effect of these two SNPs would be to shift the five-point scale by 0.3.

**TABLE 5:** SS identification numbers of the SNP, chromosome, position, negative log *p*-value, minor allele frequency (MAF), proportion of total phenotypic variation explained by the SNP (*R*^2^), allelic effect, chromosomal location and number of gene models found within a 200 kb window proximal and distal to the SNP for significant associations found from genome wide association study of *Fusarium solani* root rot disease severity and biomass in the BeanCAP Snap Bean Diversity Panel grown at the Oregon State University Vegetable Research Farm in 2014 and 2015.

Trait	SS ID No.	Chromosome	Position (bp)	-log *P.*	MAF	*R* ^2^	Effect	Chromosomal location^1^	No. gene models
Disease	ss715647578	Pv03	12,661,037	4.58	0.09	10.8	−0.15	pericentric	11
Disease	ss715646526	Pv07	34,296,485	4.51	0.37	0.9	0.09	pericentric	21
Disease	ss715639797	Pv08	32,951,182	5.24	0.23	6.2	−0.13	pericentric	7
Biomass	ss715649390	Pv10	5,677,538	4.89	0.37	14.8	−0.12	proximal	11
Disease	ss715649485	Pv10	7,910,750	4.85	0.14	7.3	−0.13	pericentric	12
Disease	ss715646318	Pv10	40,686,027	5.75	0.39	5.6	0,14	distal	35
Biomass	ss715645486	Pv11	766,814	5.35	0.22	11.3	−0.18	proximal	26

*^1^Pericentric location of a SNP is associated with low rates of recombination while proximal and distal locations are in regions of high recombination. Placement based on Supplementary Figures of physical vs. linkage map distances in Schmutz et al. (2014).*

Within a 100 kb window upstream and downstream of these SNPs, a total of 123 gene models were found across the seven regions with an average of 18 per region (Table 5). One candidate gene (peroxidase) was identified as potentially involved in disease resistance (Table 6). A total of four candidate genes were identified as potentially involved in biomass and abiotic stress tolerance, including a pentatricopeptide repeat domain and three tandem 2-alkenal reductase genes models (Table 6). Two of the three 2-alkenal reductase gene models were outside of the 100 kb window, but are included here because they were adjacent to one within the window.

**TABLE 6:** Putative candidate genes within 350 kb of the associated SNP for *Fusarium solani* root rot disease severity and plant biomass identified by genome wide association study using the BeanCAP snap bean diversity panel grown at the Oregon State University Vegetable Research Farm near Corvallis.

Chrom.	SNP position	Distance^1^	*P. vulgaris* gene model	Start	End	Gene function	References
	bp			bp			
Pv03^2^	12,661,037	74,361	Phvul.003G078600.1	12,584,313	12,586,676	Peroxidase	Ray et al., 1998
Pv10	5,677,538	76,042	Phvul.010G039100	5,753,580	5,758,499	2-alkenal reductase	Xi et al., 2015
		102,406	Phvul.010G039200	5,779,944	5,784,500	2-alkenal reductase	Xi et al., 2015
		125,106	Phvul.010G039300	5,802,644	5,808,058	2-alkenal reductase	Xi et al., 2015
Pv11	766,814	72,566	Phvul.011G010900	839,380	841,056	Pentatricopeptide repeat domain (PPR_3)	Jiang et al., 2015; Cao et al., 2020

*^1^Distance between SNP and nearest end of candidate gene. ^2^QTN on Pv03 associated with disease severity while those on Pv10 and Pv11 are associated with biomass.*

**FIGURE 2:**
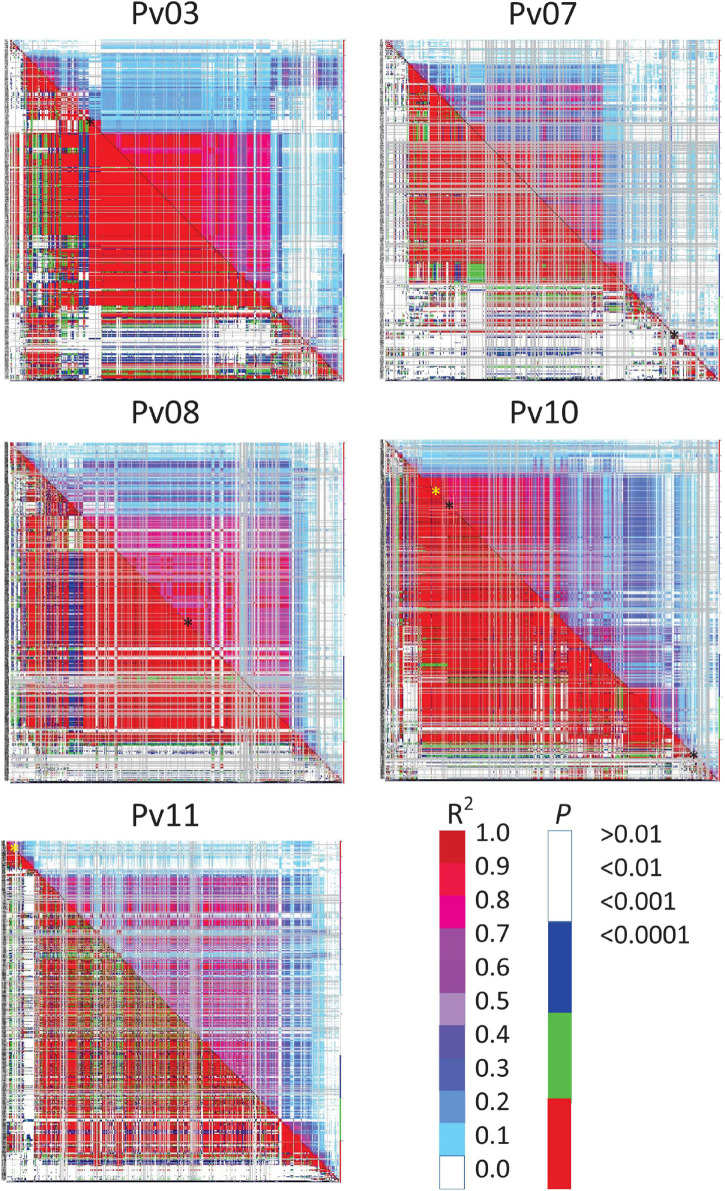
Linkage disequilibrium (LD) heat map of common bean chromosomes Pv03, 07, 08, 10, and 11 showing all possible pairwise comparisons of SNPs arranged along the chromosome. *R*^2^ values are displayed above and right of the diagonal and corresponding probabilities below and left of the diagonal. Color scales show corresponding *R*^2^ and probabilities where red for each would indicate strong and highly significant LD. SNPs associated with disease severity (black ^∗^) or biomass (yellow ^∗^) are indicated along the diagonal.

Based on a threshold of D’ or *R*^2^ ≥ 0.80 and *P* ≤ 0.01, regions of LD were identified around some significant SNPs. D’ identified extremely large blocks of LD that were on the order of 1.9–36.0 Mb for disease severity whereas *R*^2^ provided a much more conservative estimate, ranging from 150 to 679 kb (Supplementary Table 2). The LD heat map and table indicated that SNPs on Pv03, Pv07, and Pv10 for disease severity, and Pv10 for biomass were within blocks of LD (Figure 2). These ranged from 150 to 679 kb in size. The other SNPs were in LD blocks using D’ as a criterion, but not with *R*^2^ (Supplementary Table 2).

An ANOVA analysis of the trait-SNP associations supported the results of the GWAS analysis (Supplementary Figure 5). The results were uniform for years with no significant differences between years. The box plot trend supported the trait-SNP association for SNP ss715639797 with *P* = 0.08. The other SNPs for disease were significant with *P* < 0.05. The only exception was SNP ss715646526 which was not significant, and the box plots did not show any particular trend, and this was true for individual years. For biomass, ss715649390 was highly significant whereas ss715645486 was not, but it does show a trend.

### Genomic Prediction

GEBV rankings represent the general trends seen in the phenotypic data but with numerous crossovers in ranking due to the information from relatives reflected in GEBV calculations (Table 7). This can be seen in the ranking of disease severity, which has ‘Impact’, ‘Black Valentine’, ‘Widusa’, ‘NY6020-5’, and ‘Romano Gold’ as the top five most resistant lines in the phenotypic data set (data not shown) but the GEBV calculations show ‘Widusa’, ‘Impact’, ‘Double Dutch White’, ‘Booster’, and ‘Stringless French Filet’ as having the best GEBV for disease resistance (Table 7). When compared to the PCA biplot (Supplementary Figure 3), lines with the highest GEBV rankings for disease severity come from both Mesoamerican and Andean centers and provide evidence that population structure is not influencing choice of significant SNP associations.

**TABLE 7:** Genomic estimated breeding values (GEBV) calculated from BLUPs and BLUEs for the 10 highest and 10 lowest ranked lines in the BeanCAP Snap Bean Diversity Panel for *Fusarium solani* root rot disease severity, plant biomass, tap root, basal root diameter, adventitious roots, deepest and shallowest root angle.

Disease severity		Biomass		Tap root diameter		Basal root diameter		Adventitious roots		Deep root angle		Shallow root angle	
Accession	GEBV^1^	Accession	GEBV^2^	Accession	GEBV (mm)	Accession	GEBV (mm)	Accession	GEBV^3^	Accession	GEBV^4^	Accession	GEBV^4^
Widusa	2.61	Oregon 2065	4.32	Widusa	2.71	Goldrush	2.28	Widusa	1.7	Booster	48.8	Oregon Giant Pole	11.51
Impact	2.64	Idaho Refugee	4.15	Trail of Tears	2.66	Oregon 91G	2.27	Serin	1.71	Oregon 2065	48.88	Roma II	11.68
Dutch Double White	2.68	Corbett Refugee	4.04	Fortex	2.65	Gold Mine	2.26	Pole Blue Lake S7	1.72	Banga	49.08	Fortex	11.99
Booster	2.72	Gina	4	Pole Blue Lake S7	2.62	Profit	2.26	Dutch Double White	1.74	Pole Blue Lake	49.09	Ebro	12.02
Stringless French Filet	2.73	Ebro	3.97	Booster	2.56	Stringless French Filet	2.24	Impact	1.74	Serin	49.26	Magnum	12.05
Selecta	2.74	Tapia	3.92	EZ Pick	2.54	Oregon 5630	2.22	Kylian	1.76	Astun	49.3	Tapia	12.11
Pole Blue Lake	2.78	NY6020-5	3.89	Impact	2.54	Eagle	2.22	Koala	1.76	EZ pick	49.36	Astun	12.6
Oregon 2065	2.79	Coloma	3.89	Pole Blue Lake	2.53	Gina	2.22	Polder	1.77	Celtic	49.44	Idaho Refugee	12.96
Pole Blue Lake S7	2.79	Unidor	3.87	Paloma	2.51	Carson	2.21	Renegade	1.77	Stayton	49.46	Romano 118	13.01
Cherokee	2.81	Calgreen	3.82	Hayden	2.5	Summit	2.21	Pix	1.78	Redon	49.67	Cyclone	13.01
…	…	…	…	…	…	…	…	…	…	…	…	…	…
Shade	3.45	Brio	2.68	US Refugee #5	2.05	Redon	1.96	NY6020-5	2.2	Benton	59.87	Benton	18.51
Espada	3.45	Minuette	2.67	Charon	2.04	EZ Pick	1.94	Medinah	2.2	Castano	59.89	Warrior	18.66
Spartacus	3.46	Paulista	2.66	Opus	2.04	Banga	1.93	Landmark	2.2	Brio	59.91	Festina	18.68
Matador	3.49	Festina	2.65	Strike	2.04	Idaho Refugee	1.93	Benton	2.24	Shade	60.04	Zeus	18.68
Warrior	3.5	Matador	2.64	Mercury	2.04	Booster	1.93	Coloma	2.29	Summit	60.1	Matador	18.72
Titan	3.53	Palati	2.64	Dusky	2.03	Blue Peter Pole	1.91	FR-266	2.32	Carlo	60.38	Palati	18.77
Benton	3.53	Flavorsweet	2.63	Castano	2.02	Corbett Refugee	1.89	Oregon Giant Pole	2.34	Provider	60.56	Benchmark	18.88
Hercules	3.53	Dusky	2.51	Landmark	2.01	Kentucky Wonder	1.87	US Refugee #5	2.43	Stallion	60.82	Dusky	19.04
Festina	3.54	Speedy	2.4	Idaho Refugee	1.97	McCaslan No. 42	1.86	Idaho Refugee	2.55	Valentino	61.39	Castano	19.22
Seabiscuit	3.58	Embassy	2.32	Corbett Refugee	1.93	Trail of Tears	1.86	Corbett Refugee	2.6	Grenoble	61.41	Roller	19.37

*^1^Disease severity rated on a 1–5 scale where 1 is resistant and 5 is susceptible. ^2^Rated on a 1–5 scale where 1 is the least and 5 the most biomass accumulation. ^3^Rated on a 1–3 scale where 3 = most adventitious roots. ^4^Degrees from 0° to 90° where 0° represents a horizontal position.*

**FIGURE 3:**
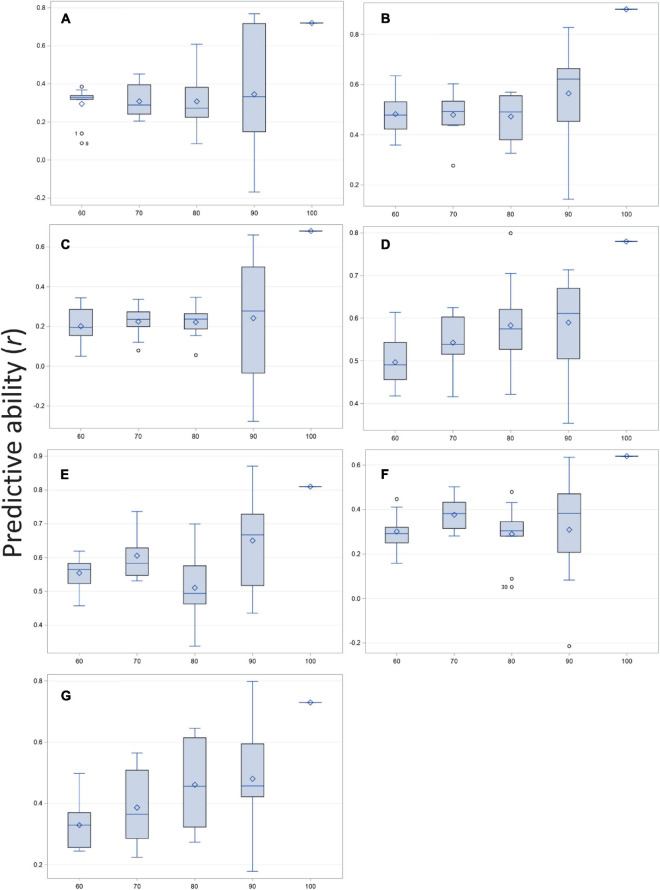
Correlation of predicted and observed values for training vs. testing populations at four ratios (60:40, 70:30, 80:20, and 90:10%) and compared with observed vs. predicted for the entire population (100%) of the BeanCAP Snap Bean Diversity Panel for *Fusarium solani* root rot disease severity and plant and root traits. Correlation coefficients were generated by rrBLUP using 10 K iterations and 10 repetitions per trait-level combination. **(A)** Adventitious roots, **(B)** Biomass, **(C)** Basal stem diameter, **(D)** Disease severity, **(E)** Deep root angle, **(F)** Shallow root angle, and **(G)** Taproot diameter.

Predicted and observed values for all traits resulted in high to moderate correlations (*r*) for disease severity, biomass, and the five root architecture traits (Figure 3 and Supplementary Table 3; 100% column in histograms and row in table). Ten thousand iterations of a cross validation with four training-testing models and replicated 10 times for each trait-model combination produced moderate to low correlations for predictive ability. The correlations that were highest under training and validation were those for disease severity, biomass and deep root angle. As size of the training population increased, mean correlation remained essentially flat (adventitious roots, basal root diameter), showed a linear increase (biomass, disease severity, and taproot diameter), or fluctuated (deep and shallow root angles). Variation about the mean of *r* was greatest at the 90% level (Figure 3 and Supplementary Table 3). Overall, standard deviations were smallest for the model with 70% training population although for biomass, 60 or 70% training models were very similar, as were 70 and 80% training models for basal root diameter. Cross-validation predictions generally were 20–40% lower than correlation among predicted and observed of the entire population. Disease severity, deep root angle and shallow root angle showed the smallest differences.

**FIGURE 4:**
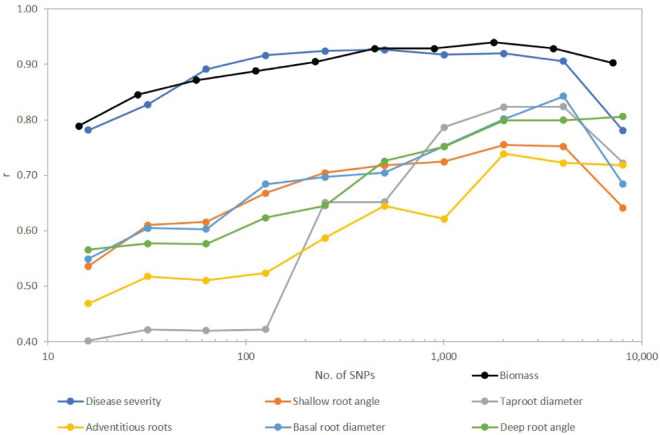
Effect of number of SNPs on predictive accuracy for *Fusarium* root rot disease severity and root traits of a snap bean diversity panel. SNPs were first filtered for MAF < 0.05, then sorted from smallest to largest *P* value and arranged in nine subsets approximately doubling in size with each step. Full set of SNPs for biomass was 7,082 while for all other traits totaled 8,036. Number of SNPs is plotted on a logarithmic (base 10) scale.

Number of SNPs retained in the model affected predictive ability. Correlation coefficients were generally lowest for the fewest significant SNPs and increased as SNPs were added to the model (Figure 4), but in most cases plateaued before declining with use of the full SNP set. The traits separated into two groups with disease severity and biomass showing relatively high correlations, and the root traits exhibiting moderate to moderately high correlations over SNP subsets. For disease severity, *r* > 0.90 was obtained with 126 SNPs, while for biomass *r* > 0.90 was obtained with 221 SNPs. Disease severity exhibited a decrease in *r* from 0.91 to 0.78 when transitioning from 4,018 to the full SNP set, and for biomass, the decrease was from 0.93 to 0.90. For root traits, most did not reach a maximum *r* until 2,009 or 4,018 SNPs were used with *r* ranging from 0.72 to 0.84. In all cases except for adventitious roots and deep root angle, *r* decreased for the full SNP set compared to half the SNPs used in the model (Figure 4).

## Discussion

Our ANOVA results showed significant year x line interaction for disease severity (*P* ≤ 0.05), biomass (*P* ≤ 0.001), adventitious roots (*P* ≤ 0.001), basal root diameter (*P* ≤ 0.05), deepest root angle (*P* ≤ 0.05), and root angle geometric mean (*P* ≤ 0.05), but no statistical significance for the remaining traits. The significant interactions for disease severity and biomass appeared to be due to differences in magnitude rather than changes in rank years as shown by moderate but highly significant correlations between years based on Spearman rank correlation (data not shown). The pattern exhibited by the replicates for disease score differed in 2014 and 2015, most likely due to differences in order of evaluation. In 2014, lines in all reps were evaluated when reaching the desired physiological stage but in 2015, reps were evaluated sequentially. In 2014, spatial variation in reps was important with the two inner reps showing more disease than the outer reps. In 2015, disease severity increased over time. Coefficient of variation (CV) was relatively low at 15 and 23 for disease severity and biomass, respectively, with other traits similar to biomass, except shallow root angle, which was had a CV of 68. The high disease pressure and consistent watering likely contributed to this uniformity across years and a low CV. Although our study could not exclude every environmental factor present in an outdoor field, these environmental factors may be both confounding but also offer the possibility of capturing complex interactions between genes and the environment that could be important to disease manifestation in a grower’s field.

Our shovelomics methodology provides a valuable window into the disease process. Our analysis showed that root angle and disease severity are positively correlated suggesting that susceptible lines had root systems oriented at a deeper angle than resistant lines (Table 4). Similar to our findings, Snapp et al. (2003) found that more lateral roots of larger diameter were associated with *Fusarium* root rot resistance. In their research on nutrient foraging, Lynch and Brown (2001) emphasized that a plant with exclusively deep root angles is exploring a smaller amount of soil than a plant with either a shallow or a range of root angles. The beans with shallower root systems may have been able to access a greater soil volume. Another possible explanation for the effect observed in this study is that the upper layer of soil had superior drainage, which reduced infection by root rot. There may be a tradeoff between disease resistance and drought tolerance with regard to root angle. Drought tolerant plants will likely have roots exploring greater depths of soil.

The negative correlation of disease severity with aboveground biomass, basal root diameter, and taproot diameter, indicated that resistant cultivars had greater aboveground biomass and larger root diameter than susceptible cultivars (Table 4). The positive correlation of disease severity with adventitious roots, shallowest root angle and deepest root angle indicated that cultivars with fewer adventitious roots and shallower root angles were associated with less disease. For aboveground biomass, basal root diameter and taproot diameter, the highly positive correlation indicated that the magnitude of the three size measurements maintained a constant relationship across lines. Aboveground biomass and taproot diameter were negatively correlated with shallowest and deepest root angle, meaning that larger plants had shallower root systems. Positively correlated shallowest and deepest root angles indicated that regardless of the orientation of the root system, the span of the soil profile that it accessed remained constant.

Disease severity, biomass, and adventitious roots had higher heritability than the other shovelomics traits, such as root angle measurements. The heritability value for disease resistance is within the range of values measured by most previous researchers. Hagerty et al. (2015) obtained *h*^2^ of ∼0.20 and Mukankusi et al. (2011) reported heritability of 0.38–0.45 for root rot resistance. In contrast, Kamfwa et al. (2013) found higher heritabilities of 0.86–0.99. The heritability for aboveground biomass found in this study also corresponds to previously reported values. Shenkut and Brick (2003) found a range from 0.60 to 0.70. Navarro et al. (2008) reported values of 0.77–0.91 for heritability of biomass, based on measurements of dry weight, which implies that our categorical rating system did not greatly inflate heritability values. The high heritability values imply that simple selection strategies on these traits would be effective.

The high heritabilities of disease severity and biomass are consistent with the high correlational accuracy of these two traits in genomic prediction and the significant results in GWAS. These two traits were also negatively correlated with a high statistical significance (Table 4) indicating the possibility that disease stressed plants were generating less biomass. Nevertheless, these traits are not entirely overlapping and the negative correlation may be partly coincidental and not causal because GWAS analysis identified distinct SNP markers for disease severity and biomass.

The lack of GWAS results for five of seven traits is notable. There may be confounding factors associated with measuring traits under disease pressure. As noted already, the other traits had lower heritabilities that may also explain the difference. Moreover, the Bonferroni and bootstrap thresholds utilized in this study are very conservative. Additionally, increasing the population size and/or number of SNPs would have led to greater precision and a greater likelihood of detecting significant associations.

The SNPs identified by our GWAS analysis did not clearly overlap with any previously identified SNP from GWAS analysis or biparental analysis of root rot organisms (Hagerty et al., 2015; Oladzad et al., 2019; Dramadri et al., 2020; Zitnick-Anderson et al., 2020). We identified one candidate gene related to plant defense within the immediate vicinity of an associated SNP (Table 6). Peroxidases are involved in the final steps of the biochemical pathway leading to lignification, which directly interferes with pathogen invasion (Ray et al., 1998).

From our studies and those of others (Hagerty et al., 2015; Nakedde et al., 2016; Wang et al., 2018; Zitnick-Anderson et al., 2020), there is strong evidence that *F. solani* resistance in common bean is polygenic with many genes with small effect being involved. One interesting finding is the lack of commonality of resistance QTL among the different studies where genome location can be compared. This would support the idea of polygenic resistance based on genes that are not considered classical resistance genes. Given the level of resistance in some lines in our diversity panel, it is possible to achieve relatively high levels of resistance with the right gene combination, which appears to confer broad-spectrum resistance to different *Fusarium* isolates. While virulence may vary among isolates, there does not appear to be a pathogen race structure. As a case in point, the resistance in FR-266 was relatively effective to *Fusarium* isolates endemic to Michigan (Schneider et al., 2001; Snapp et al., 2003), whereas we found this genotype to be moderately resistant against our field isolates in Oregon, implying that Oregon isolates were more virulent. However, in both cases, resistance was quantitative with no clear major QTL.

Where host and pathogen are coevolving under antagonist selection, the prediction is resistance genes would evolve in concert and tend accumulate in large haplotype blocks in low recombining genomic regions (Ravinet et al., 2017). Our findings lend support to that idea in that of the five SNPs associated with disease severity, four were located in low-recombination, gene-sparse pericentric regions and only one was located distally on Pv10 in a high-recombination region (Table 5). Both SNPs associated with biomass were in high-recombination regions located proximally on their respective chromosomes.

Linkage disequilibrium heatmaps (Figure 2 and Supplementary Table 2) provide a more detailed examination of low recombination blocks in relations to chromosomal location, and are in partial agreement with low recombination regions identified in Table 5. Visually, Figure 2 aligns with categories in Table 5. One discrepancy between Table 5 and Supplementary Table 2 was for the SNP associated with disease severity on Pv08, where the SNP clearly resides in a region of low recombination (based on physical vs. cM biplots in Schmutz et al., 2014), however, an LD block for this region was essentially non-existent based on an *R*^2^ cutoff of 0.80. The heatmap (Figure 2) does show moderate to high LD in this region. The second discrepancy was for a SNP on Pv10 associated with biomass. This SNP is located proximally, but had a sizable LD block of 421 kb. Pv10 is acrocentric and the SNP is located in the short arm, which have reduced recombination (see Supplementary Figure 13 in Schmutz et al., 2014).

A further implication of the location of most resistance associated SNPs in low recombination regions is that marker assisted selection would be at best, inefficient and at worst, ineffective because of the large non-recombinant blocks of genes. This provides further support for prioritizing genomic selection over QTL mapping and marker assisted selection of individual QTL.

The biomass candidate genes were identified through their known effects on biomass but also their effects on abiotic stress tolerance because disease pressure can induce drought stress in affected bean plants through the loss of their roots to disease. A pentatricopeptide repeat (PPR) domain candidate gene was found in the vicinity of SNP ss715645486. PPR domains have been implicated in an increase of biomass in a study of *Paulownia* trees (Cao et al., 2020), and are also implicated in drought stress tolerance (Jiang et al., 2015). The three tandem duplicate genes of 2-alkenal reductase in the vicinity of SNP ss715649390 are also implicated in increased biomass and improved drought tolerance in a study of transgenic tobacco plants (Xi et al., 2015).

Are there tradeoffs between *Fusarium* resistance and abiotic stress tolerances? Burke and Miller (1983) extensively analyzed the interactions of *Fusarium* root rot with various cultural practices that can affect the development of disease. Their findings were that anything that constricts the root system (such as cold soils and compaction) will exacerbate disease development. Intermittent drought stress combined with these factors restricting root growth will further increase disease pressure. Excess soil moisture even if it is intermittent and of short duration will prevent oxygen diffusion to the roots and further inhibits root growth. High population densities also tend to increase root rot. Previously bred *Fusarium* root rot resistant dry bean cultivars tended to tolerate cold soils, drought and compaction better than susceptible cultivars, but in waterlogged soils, resistance was defeated. In the present study, there does not appear to be a tradeoff among these traits with one exception: the correlation of shallow root angle with disease resistance, which might lead to less drought tolerant plants. Correlation is not causation so this supposition would need to be tested and could be carried out by subjecting the snap bean diversity panel to drought as well as other forms of abiotic stress. On the other hand, nutrient use efficiency, especially for phosphorous (P), is associated with shallow root systems (Lynch and Brown, 2001). Breeding for P use efficiency would not likely impact root rot resistance and vice versa.

The multiple associated SNPs detected for disease severity with low *R*^2^ values and their non-overlap with numerous SNPs detected for root rot in other studies strongly suggests that root rot resistance is highly polygenic in nature with numerous loci of low effect. This further supports the notion that genomic selection, which fully utilizes all SNPs, may be a better method to breed for root rot disease resistance in snap bean than identifying a small number of loci in GWAS and applying marker assisted selection to those loci.

Optimum ratio of training to testing populations for achieving the highest repeatable predictive ability was 70:30% training:validation for most traits. This level is within the range of what has been found for other studies of genomic prediction in common bean (Keller et al., 2020; Diaz L. M. et al., 2021; Diaz S. et al., 2021; Shi et al., 2021). At 90% training population, the highest average predictabilities as measured by *r* were achieved, but standard deviations were much larger, leading to less certainty in whether a prediction was accurate. Shi et al. (2021) reported that training sets >80% can lead to large variation associated with too small a validation set.

In evaluating the influence of the number of SNPs on prediction accuracy, it was curious that for most traits, the full set of SNPs used in our model had lower predictive accuracy compared to a reduced number of SNPs. Studies in bean and other crops have generally shown a positive correlation between number of SNPs and predictive accuracy (Spindel et al., 2016; Liu et al., 2018; Wen et al., 2019; Keller et al., 2020; Thistlethwaite et al., 2020; Arenas et al., 2021; Shi et al., 2021). These studies do differ in how many SNPs were used and in how they were selected for each subset, but the overall trends were similar. Some studies have observed decreases in predictive accuracy at various SNP levels. Thistlethwaite et al. (2020) observed a drop at around 10,000 SNPs before rising again. Arenas et al. (2021) observed a dip at around 1,000–1,500 SNPs for four traits. In our study, disease severity and biomass could be modeled with a high degree of accuracy (*r* > 0.90) with relatively few (126–221) SNPs. In contrast, root traits were best modeled with one-half to one-quarter of the full SNP data set. Other studies have shown that genomic prediction models that incorporate GWAS can improve accuracy in breeding programs (Spindel et al., 2016). Shi et al. (2021) found the highest predictive accuracy when 20 SNPs derived from GWAS were used. Our selection of 14 (biomass) and 16 (disease severity) most highly significant SNPs had among the lowest predictive accuracies. Our results reinforce the idea that resistance to *Fusarium* root rot is polygenic and requires many genes to achieve the highest levels of resistance.

One of the important questions in GWAS has been how to account for the “missing heritability” in such studies (Manolio et al., 2009). Relative to the heritability estimates based on phenotypic and genotypic variances, the amount of variation explained by significant SNP associations is small, and the cumulative effect of all associations in the model does not always approximate classical measures of heritability. This is particularly true where QTL have small individual effect. In the present study, the *h*^2^ estimate based on genotypic and phenotypic variances was relatively high 0.74 for disease severity and 0.75 for biomass (Table 2) while the cumulative *R*^2^ for the SNPs associated with these traits ranged from 0.26 to 0.31. This implies that either *h*^2^ is overestimated, or that GWAS may be missing medium- and low-effect associations. Relaxing our cutoff for identifying SNP associations could lead to the identification of additional associations, but increasing number of genotypes and/or markers would provide the greatest possibility of accurately detecting additional associations.

One piece of the missing heritability may be conditioned by genetic variability in the phenolic/flavonoid biosynthetic pathway. Flavonoids and phenolics have been shown to possess antimicrobial properties which have been associated with resistance to root rots (Hagerty et al., 2015; Cirak and Myers, 2021). One line (‘Cherokee’) from those with the highest rank for GEBV for disease severity had colored seeds and flowers, while none of the lowest ranked lines were colored (Table 7 and Supplementary Table 1 in Kleintop et al., 2016). The SBDP has been evaluated for total phenolic content (TPC) of pods (Kleintop et al., 2016), which can serve as a proxy for phenolics and flavonoids distributed in other plant parts. The 10 lines with lowest GEBV values for disease severity had relatively higher TPC than did the 10 lines with the highest GEBV (mean of 0.52 vs. 0.40 mg g^–1^ FW gallic acid equivalents). Disease severity and GEBV for disease severity were negatively correlated with TPC (*r* = -0.18, *P* = 0.03 and *r* = -0.23, *P* = 0.005, respectively). Myers et al. (2019) conducted a GWAS for TPC in pods of the SBDP and when we compared those results to the current findings, we did not find any overlap in regions of significant SNPs for disease severity or biomass. These results are compatible with the idea that phenolics do play a role in root rot resistance although it is not a major one.

To achieve acceptable processing quality, most contemporary snap bean cultivars are white-seeded, which eliminates anthocyanins and flavonols from the pods. If we had found a strong relationship between TPC and disease severity, those associations with pigment production would not be useful in a breeding program. Although lines varied for total TPC, all but one was white-seeded (preventing anthocyanin accumulation in the pods) and thus do not present barriers to use in a breeding program for root rot resistance.

In common bean, geographic origin and population structure have been shown to be an important influence on genetic variation in wild and landrace beans (Blair et al., 2012; Cortés et al., 2018). With the BeanCAP snap bean diversity panel, we do not expect associations that might be related to demography since snap bean origins are not associated with a particular place. However, snap beans do appear to have been secondarily derived from dry beans, and indirectly from the two centers of domestication, possibly with several independent events, and have retained some genetic signature of their origins (Wallace et al., 2018). Derivation has been followed by substantial admixing, which has reduced distinct associations with centers of domestication and has produced more of a clinal variation across the diversity panel. Population structure could result in spurious marker – trait associations; however, structure was accounted for in the FarmCPU model, and we did not see any pattern between disease severity GEBVs and location on the PCA biplot.

This research builds on prior work on *Fusarium* root rot resistance in common bean and will give snap bean breeders additional tools to dissect and manipulate resistance to *Fusarium* root rot in snap beans. The heritabilities give information on the expected gain from selection that could be achieved. The correlations among disease and root traits provide valuable information on the root architecture necessary to develop resistant lines. The GWAS analysis provides additional markers to a growing number associated with resistance. The genomic predictions identify individual lines with genetic merit worth pursuing by utilizing the totality of marker effects. Future research could include a more detailed investigation root trait associations with biotic and abiotic stress tolerance, combine snap bean data with dry bean for a meta-GWAS, and development of a MAGIC population (Cavanagh et al., 2008) to facilitate recombination of SNP associations into a common snap bean background.

## Data Availability Statement

The datasets presented in this study can be found in online repositories. The names of the repository/repositories and accession number(s) can be found below: ScholarsArchive@OSU: https: // ir . library . oregonstate . edu / concern / datasets / m900p1589 for SNP data; Bean CAP Snap Bean Diversity Panel passport data are also available: https://ir.library.oregonstate.edu/concern/datasets/2n49t8455.

## Author Contributions

JM conceived of the field study. AH designed and collected all the data on the measured traits and contributed to significant portions of the writing through her master’s thesis. AH and JM performed the analysis in SAS for the ANOVA, correlations, and heritabilities. LW condensed and edited the manuscript of AH, contributed significantly to the writing, performed the GWAS, rrBLUP, ANOVA, and QQ plots of residuals in R, and created LD heat maps and PCA in TASSEL. JM and LW performed genomic prediction analyses, and edited and contributed to all parts of the writing and analysis. All authors contributed to the article and approved the submitted version.

## Conflict of Interest

The authors declare that the research was conducted in the absence of any commercial or financial relationships that could be construed as a potential conflict of interest.

## Publisher’s Note

All claims expressed in this article are solely those of the authors and do not necessarily represent those of their affiliated organizations, or those of the publisher, the editors and the reviewers. Any product that may be evaluated in this article, or claim that may be made by its manufacturer, is not guaranteed or endorsed by the publisher.

## Footnotes

1
https://www.maizegenetics.net/tassel
