# Supplementary material for: Associated SNPs, Heritabilities, Trait Correlations, and Genomic Breeding Values for Resistance in Snap Beans (Phaseolus vulgaris L.) to Root Rot Caused by Fusarium solani (Mart.) f. sp. phaseoli (Burkholder)
Source: Front Plant Sci. 2021 Sep 28;12:697615. doi: 10.3389/fpls.2021.697615 (PMC8507974; doi:10.3389/fpls.2021.697615)
Supplement: Supplementary file 1 [file Data_Sheet_1.pdf]

Associated SNPs, heritabilities, trait correlations, and genomic breeding values for resistance in snap beans (*Phaseolus vulgaris* L.) to root rot caused by *Fusarium solani* (Mart.) f. sp. *phaseoli* (Burkholder)

Supplementary Material

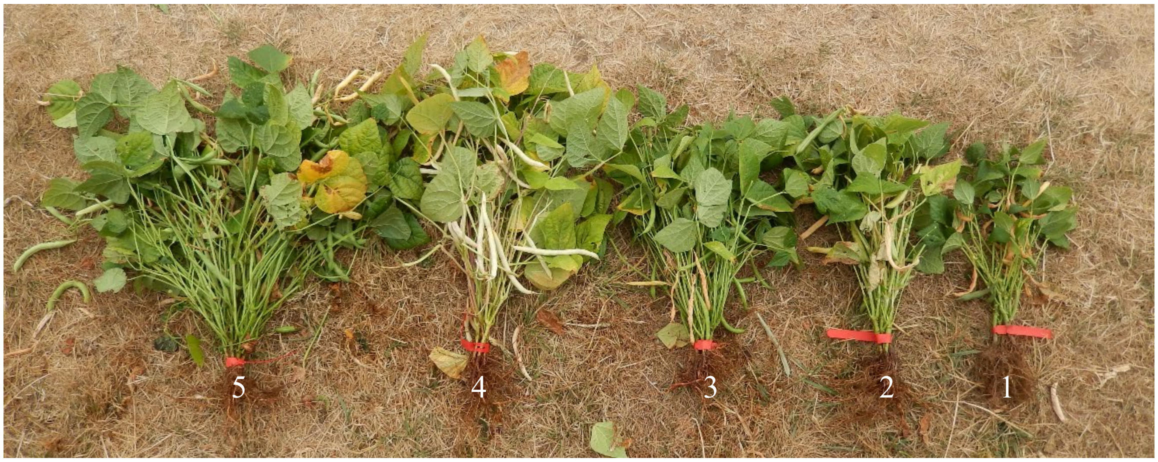

Supplementary Figure 1. Aboveground biomass of five-plant samples of common bean in root rot trials in 2015 in Oregon. Scale 1-5, right-left.

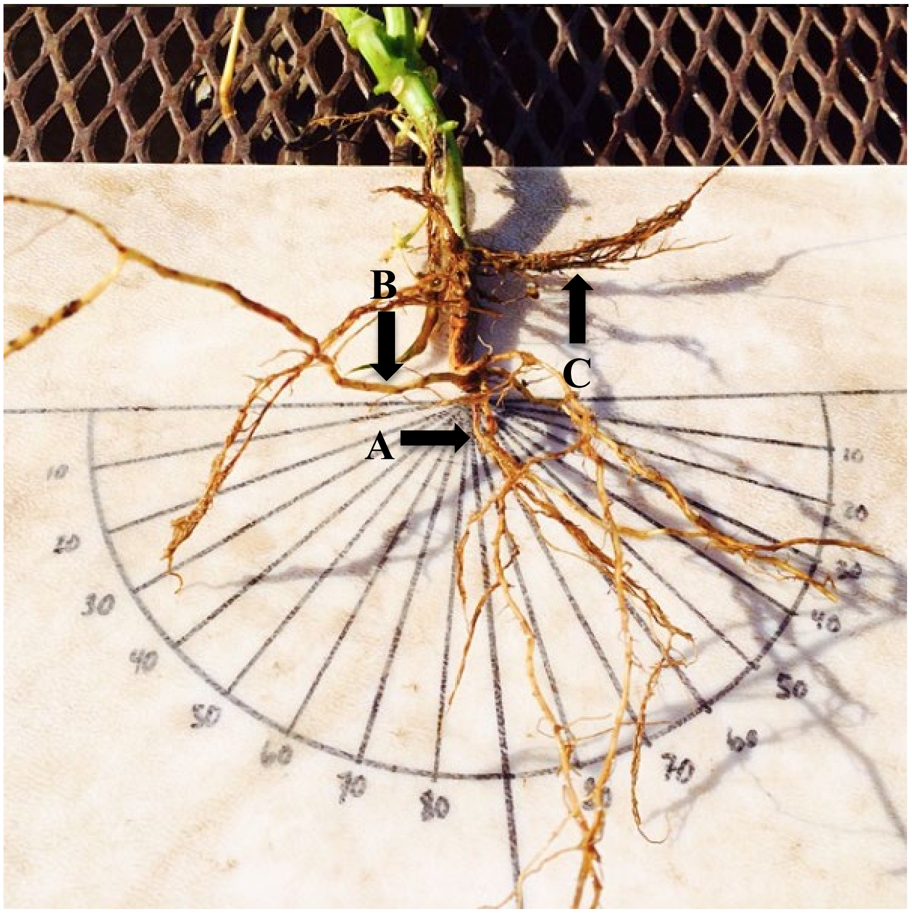

Supplementary Figure 2. Protractor device used to measure root angles in the Bean CAP Snap Bean Diversity Panel. A = taproot, B = basal roots, C = adventitious roots.

This is a provisional file, not the final typeset article

frontiers

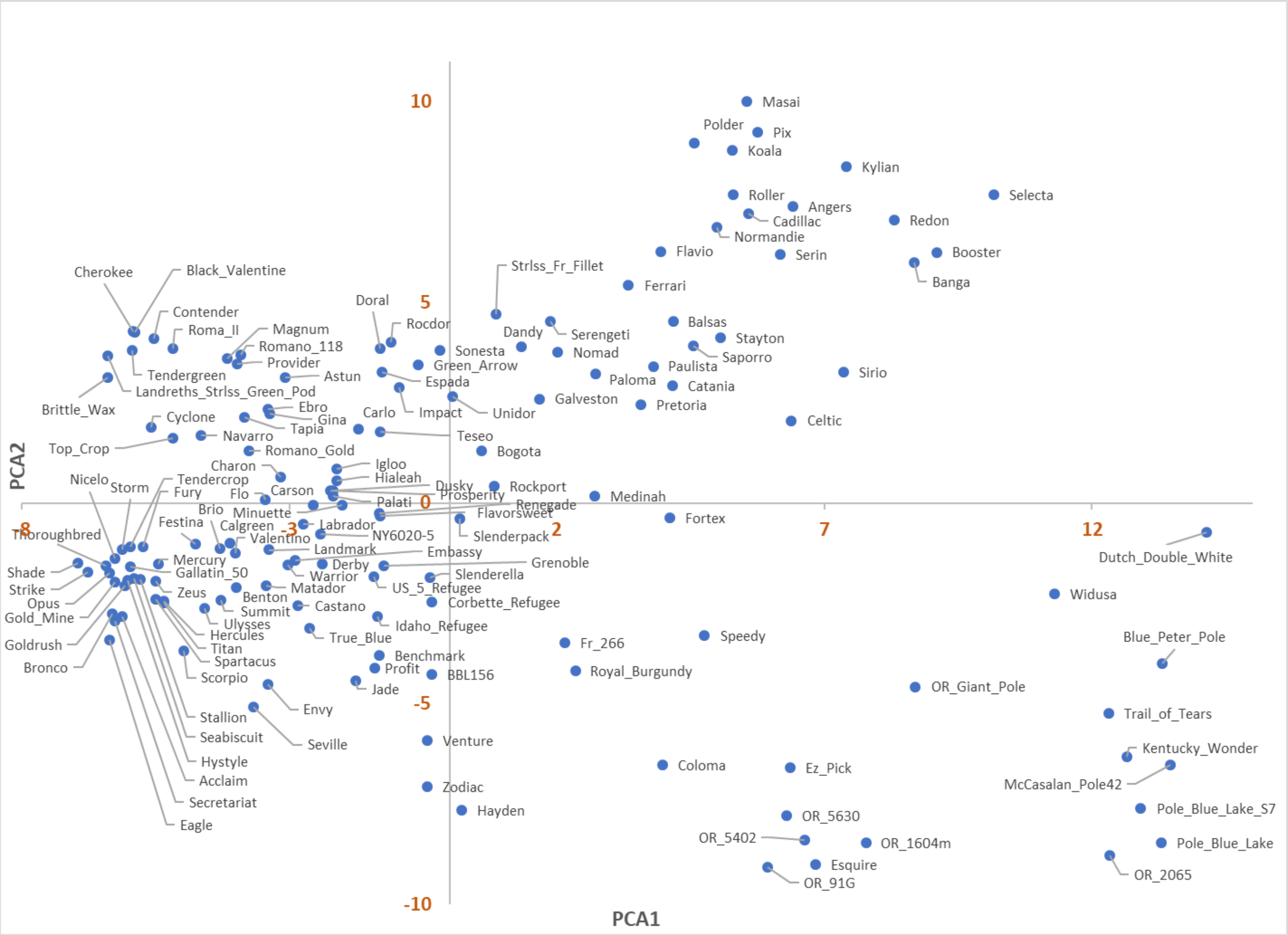

Supplementary Figure 3. Biplot showing Principle Components axes 1 and 2 for the BeanCAP Snap Bean Diversity Panel genotyped with 10.6K SNPs. PCA1 explains 22% of the variation and PCA2 accounts for an additional 11%. The first PC axis separates Andean from Mesoamerican ancestry but rather than distinct groups, there is a gradient along the axis. The second PC axis primarily differentiates within the Mesoamerican group between European and American derived lines, and between ancestral and contemporary snap beans for Andean types.

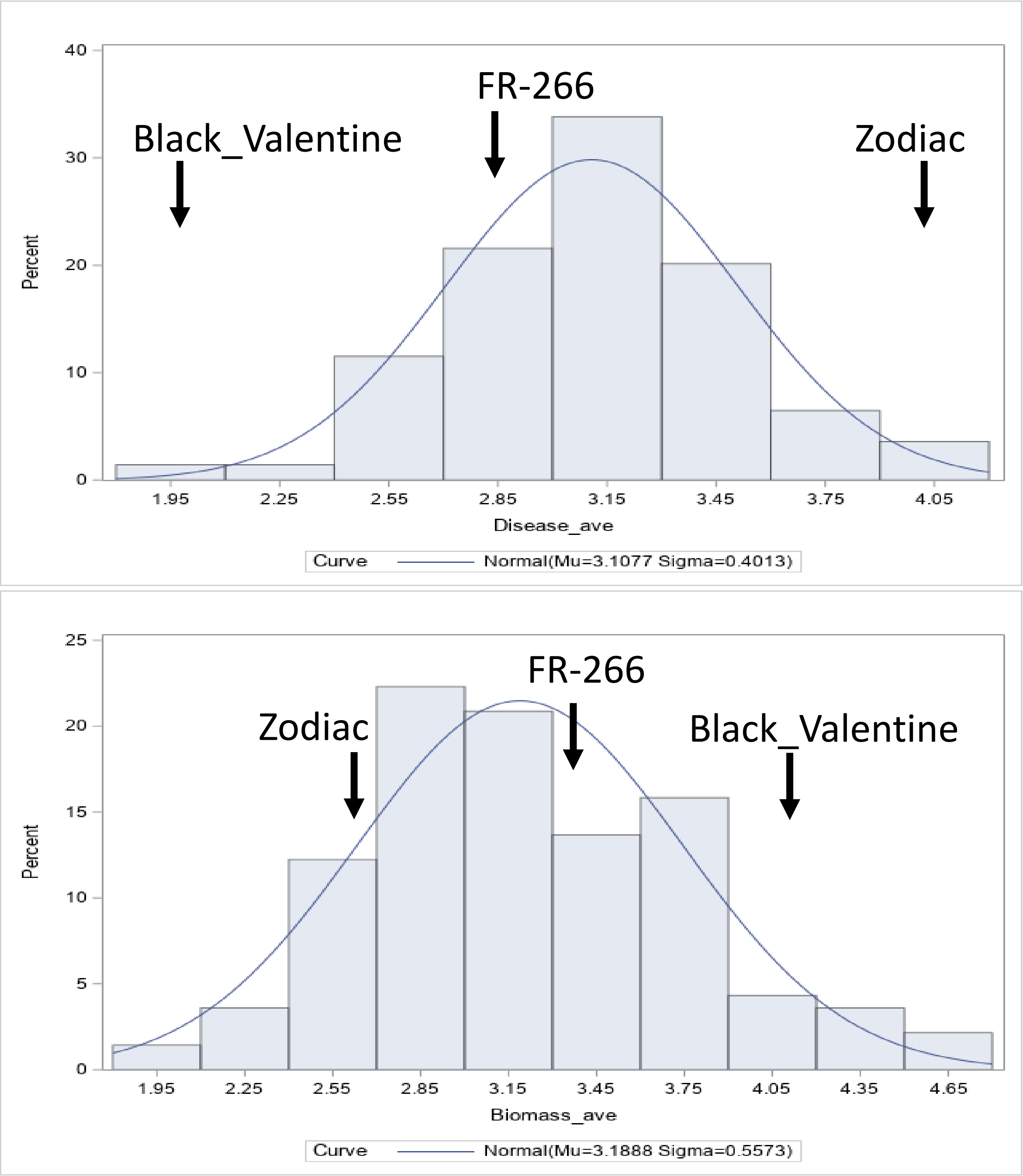

Supplementary Figure 4. Histogram and normality plots for phenotypic performance for *Fusarium solani* root rot reaction and biomass of the BeanCAP Snap Bean Diversity Panel grown at the Vegetable Research Farm. Means of replicates and years combined are plotted. Rating scales of 1 – 5 were used for both traits. Only determinate bush type cultivars are shown for biomass. ‘Black Valentine’ is a root rot resistant/high biomass cultivar and ‘Zodiac’ is a root rot susceptible/low biomass cultivar. ‘FR-266’ is a moderately resistant snap bean experimental line.

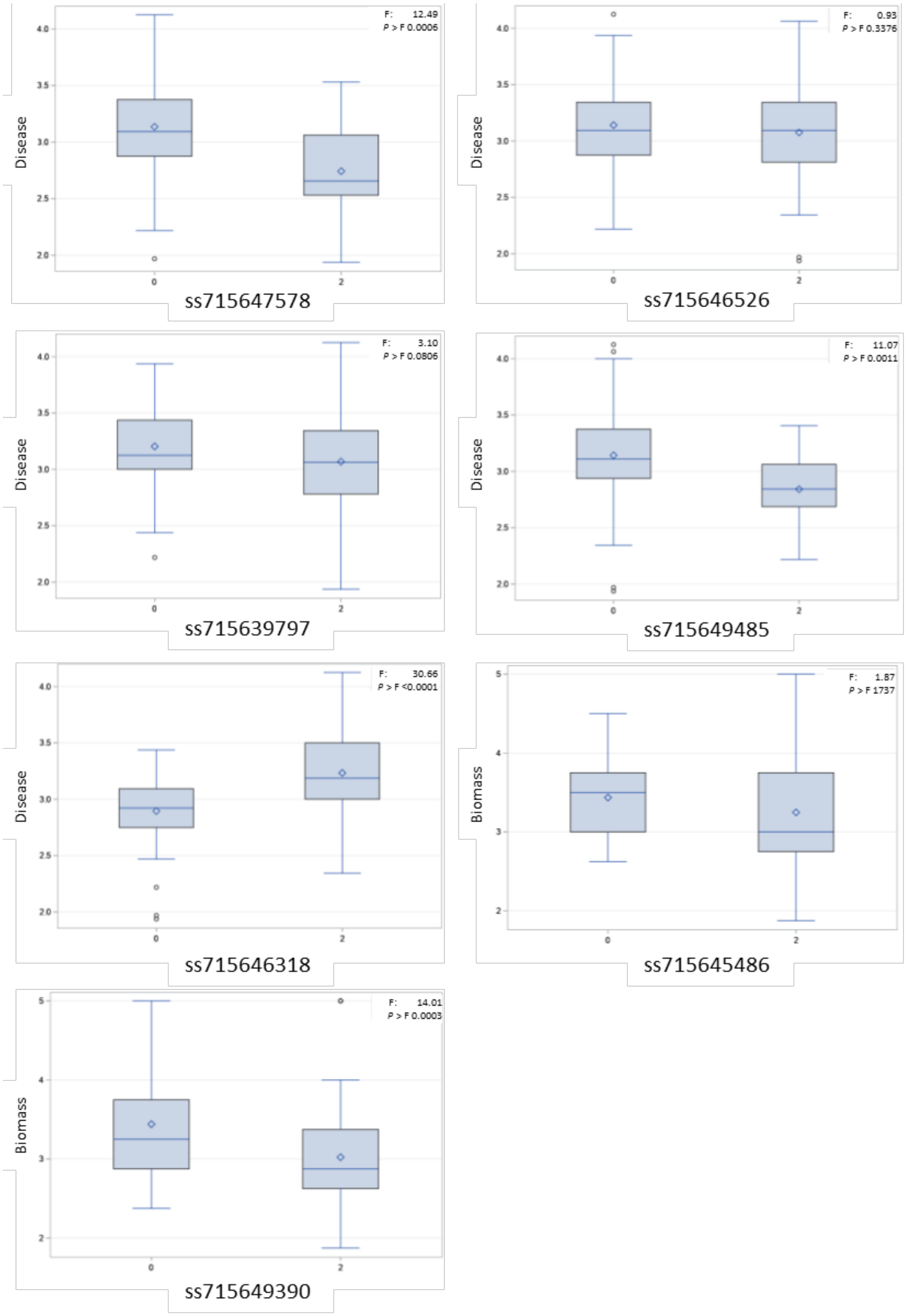

Supplementary Figure 5. Box and whiskers plots based on one-way ANOVAs of SNPs associated with disease severity or biomass from a GWAS of the BeanCAP snap bean diversity panel grown and phenotyped at the OSU Vegetable Research Farm.

Supplementary Table 1. Published Quantitative Trait Loci (QTL) for root rot in common bean, including population, type of molecular marker, *Phaseolus vulgaris* chromosome, percent total genetic variation explained (R^2), LOD score and pathogen species. "-" = Data not available.

Authors	Population type	Population	QTL associated with	Chrom.	No. of QTL	R^2	LOD	Root Rot Pathogen
Schneider et al. 2001	biparental	Montcalm x FR-266 & Isles x FR-266	RAPD	01, 02, 03, 04, 05, 06, 07	15	0.05-0.31	-	*F. solani*
Chowdhury et al. 2002	biparental	AC Compass x NY2114-12	RAPD	.	2	0.20-0.30	5-8	*F. solani*
Roman-Aviles & Kelly 2005	biparental	Red Hawk x Negro San Luis & C97407 x Negro San Luis	RAPD	01, 05, 07, 08, 09	10	0.07-0.53	2.4-15.7	*F. solani*
Navarro et al. 2008	biparental	Eagle x Puebla 152	RAPD	03, 06, 07	5	0.25-0.49	2.6-11.3	*Pythium ultimum* & *Aphanomyces euteiches*
Kamfwa et al., 2013	biparental	K132 × MLB-49-89A; K20 × MLB-49-89A	SSR	03	1	0.34	6.1	*F. solani*
Hagerty et al. 2015	biparental	OSU5446 x RR6950	SNP	02, 04, 06<br>03, 07	3<br>2	0.05-0.15<br>0.09-0.22	6.7-9.3<br>6.2-11.5	*A. euteiches*<br>*F. solani*
Nakedde et al., 2016	biparental	Puebla 152 x Zorro	SNP	05	1	0.10	3.2	*F. solani*
Wang et al., 2018	biparental	CAL96 x MLB-49-89A	SNP	02	1	0.09-0.10	2.6-3.9	*F. brasiliense*
Oladzad et al. 2019	GWAS	Andean Diversity Panel<br>Middle American Diversity Panel	SNP	02<br>06, 08, 10	1<br>3	0.12<br>0.05-0.08	-<br>-	*Rhizoctonia solani*

Authors	Population type	Population	QTL associated with	Chrom.	No. of QTL	R^2	LOD	Root Rot Pathogen
Dramadri et al., 2020	GWAS	Andean Diversity Panel	SNP	01, 02, 04, 05, 09	16	0.29-0.39	-	*P. ultimum*
Zitnick-Anderson et al., 2020	GWAS	Andean Diversity Panel	SNP	01 02, 03, 04, 07, 08, 09, 11	16	0.09-0.15	-	*F. solani*
		Middle American Diversity Panel		01, 03, 04, 07, 08	7	0.07-0.10	-	
Paulino et al., 2021	GWAS	Middle American Diversity Panel	SNP	01, 03, 04, 05, 07, 10, 11	2	0.09-0.64	-	*F. oxysporum*

Supplementary Table 2. Extent of linkage disequilibrium (LD) around SNPs associated with *Fusarium solani* disease reaction or biomass of a diversity panel of snap beans. Cutoff for LD based on a D' or R^2 of 0.8 and $P \leq 0.01$.

				D'			R^2		
Trait	SS ID No.	Chromosome	Position	Proximal	Distal	Size of LD block	Proximal	Distal	Size of LD block
			bp						
Disease	ss715647578	Pv03	12,661,037	11,799,126	32,624,773	20,825,647	12,640,644	13,319,393	678,749
Disease	ss715646526	Pv07	34,296,485	28,548,135	35,840,739	7,292,604	34,080,347	34,296,485	216,138
Disease	ss715639797	Pv08	32,951,182	18,361,451	54,388,710	36,027,259	-	-	0
Biomass	ss715649390	Pv10	5,677,538	4,962,514	7,431,711	2,469,197	5,495,323	5,916,721	421,398
Disease	ss715649485	Pv10	7,910,750	6,170,351	8,060,803	1,890,452	7,910,750	8,060,803	150,053
Disease	ss715646318	Pv10	40,686,027	38,845,021	41,217,646	2,372,625	-	-	0
Biomass	ss715645486	Pv11	766,814	716,937	766,814	49,877	-	-	0

This is a provisional file, not the final typeset article

Supplementary Table 3. Mean and standard deviation (SD) for predictive ability (r) at four ratios of training to testing populations compared to observed and predicted correlation for the entire population for seven root and *Fusarium* root rot traits of the BeanCAP Snap Bean Diversity Panel.

Training population (%)	Adventitious roots		Biomass		Basal root diameter		Disease severity		Deep root angle		Shallow root angle		Tap root diameter	
	Mean	SD	Mean	SD	Mean	SD	Mean	SD	Mean	SD	Mean	SD	Mean	SD
60	0.295	0.098	0.483	0.082	0.202	0.091	0.497	0.062	0.554	0.046	0.301	0.082	0.329	0.077
70	0.308	0.090	0.480	0.087	0.225	0.077	0.543	0.062	0.606	0.071	0.376	0.070	0.387	0.118
80	0.308	0.162	0.473	0.089	0.222	0.079	0.583	0.114	0.511	0.106	0.290	0.133	0.461	0.145
90	0.345	0.348	0.565	0.197	0.242	0.309	0.590	0.113	0.650	0.148	0.309	0.244	0.481	0.170
100	0.720	-	0.900	-	0.680	-	0.780	-	0.810	-	0.640	-	0.730	-
